# c-Src Recruitment is Involved in c-MET-Mediated Malignant Behaviour of NT2D1 Non-Seminoma Cells

**DOI:** 10.3390/ijms20020320

**Published:** 2019-01-14

**Authors:** Erica Leonetti, Luisa Gesualdi, Katia Corano Scheri, Simona Dinicola, Luigi Fattore, Maria Grazia Masiello, Alessandra Cucina, Rita Mancini, Mariano Bizzarri, Giulia Ricci, Angela Catizone

**Affiliations:** 1 Department of Anatomy, Histology, Forensic-Medicine and Orthopedics, “Sapienza” University of Rome, 00161 Rome, Italy; erica.leonetti@uniroma1.it (E.L.); luisa.gesualdi@uniroma1.it (L.G.); katia.coranoscheri@uniroma1.it (K.C.S.); angela.catizone@uniroma1.it (A.C.); 2 Department of Surgery “Pietro Valdoni”, “Sapienza” University of Rome, 00161 Rome, Italy; simona.dinicola@uniroma1.it (S.D.); mariagrazia.masiello@uniroma1.it (M.G.M.); alessandra.cucina@uniroma1.it (A.C.); 3 Department of Experimental Medicine, “Sapienza” University of Rome, 00161 Rome, Italy; mariano.bizzarri@uniroma1.it; 4 IRCCS, Regina Elena National Cancer Institute, 00144 Rome, Italy; luigifattore1985@gmail.com; 5 Azienda Policlinico Umberto I, 00161 Rome, Italy; 6 Department of Molecular and Clinical Medicine, “Sapienza” University of Rome, 00189 Rome, Italy; rita.mancini@uniroma1.it; 7 Systems Biology Group Lab, 00161 Rome, Italy; 8 Department of Experimental Medicine, Università degli Studi della Campania “Luigi Vanvitelli”, 80138 Naples, Italy

**Keywords:** TGCTs, c-MET, HGF, c-MET inhibitors, c-Src, Src inhibitors, cancer therapy

## Abstract

c-MET pathway over-activation is the signature of malignancy acquisition or chemotherapy resistance of many cancers. We recently demonstrated that type II Testicular Germ Cell Tumours (TGCTs) express c-MET receptor. In particular, we elucidated that the non-seminoma lesions express c-MET protein at higher level, compared with the seminoma ones. In line with this observation, NTERA-2 clone D1 (NT2D1) non-seminoma cells increase their proliferation, migration and invasion in response to Hepatocyte Growth Factor (HGF). One of the well-known adaptor-proteins belonging to c-MET signaling cascade is c-Src. Activation of c-Src is related to the increase of aggressiveness of many cancers. For this reason, we focused on the role of c-Src in c-MET-triggered and HGF-dependent NT2D1 cell activities. In the present paper, we have elucidated that this adaptor-protein is involved in HGF-dependent NT2D1 cell proliferation, migration and invasion, since Src inhibitor-1 administration abrogates these responses. Despite these biological evidences western blot analyses have not revealed the increase of c-Src activation because of HGF administration. However, notably, immunofluorescence analyses revealed that cytoplasmic and membrane-associated localization of c-Src shifted to the nuclear compartment after HGF stimulation. These results shed new light in the modality of HGF-dependent c-Src recruitment, and put the basis for novel investigations on the relationship between c-Src, and TGCT aggressiveness.

## 1. Introduction

Type II Testicular Germ Cell Tumours (TGCTs) (seminomas and non-seminomas) are the most common testicular tumours amongst adolescents and young adults, whose incidence is constantly increasing especially in western countries [1]. Even if these cancers are characterized mainly by a good prognosis, since they are extraordinarily chemo- and radio-sensitive [2,3,4], in a small percentage of cases a cisplatin-resistance exists, making cure difficult [5]. The development of Type II TGCTs is peculiar: they origin, in fact, from a common precursor lesion, the Germ Cell Neoplasia In Situ (GCNIS), which in turn arises from the block of differentiation of Primordial Germ Cells/gonocytes [6,7,8]. This event is due, according to the most accredited theory, to a combination of genetic and epigenetic aberrations with micro-environmental cues that, jointly, lead to the disease [9]. For this reason, these tumours can be considered at the crossroad of developmental and neoplastic processes [10], and represent an interesting model to study the relationships between these two phenomena. Notably, the molecular features of the “genviroment” that cause the acquisition of testicular germ cell neoplastic behaviour has been only partially elucidated [11]. One of the signal molecules constantly present in the testis, from early embryonic development to adult stage, is the Hepatocyte Growth Factor (HGF) [12,13,14,15,16,17,18]. This molecule is able to bind to its unique receptor, c-MET [19], influencing many activities of testicular somatic and germ cells in both humans and rodents [12,13,14,15,16,17,18]. It is well known that HGF/c-Met system regulates, in other organs, both embryonic development and cancer progression [20]. This is one of the main reasons that made us study the functions of the HGF/c-MET system in these cancer types, in which, as previously mentioned, cancer development arises from embryonic development deregulation. Recently, our group demonstrated that c-MET receptor is expressed in biopsies derived from patients affected by type II TGCTs, and in some TGCT-derived cell lines that are TCam-2, NCCIT and NT2D1 cells [21]. Moreover, in the same paper we reported that the non-seminomatous lesions express c-MET receptor at higher levels compared with the GCNIS and seminoma counterparts. In line with these clinical data, the non-seminoma derived NT2D1 cells exhibit the highest sensitivity to HGF administration: these cells respond to HGF increasing their malignant behaviour in terms of proliferation, chemo-attraction and invasiveness [21]. These observations lead us conclude that the HGF-mediated c-MET activation could be involved in the acquisition of malignancy of type II TGCTs. It is of fair notice that the relevance of c-MET/HGF system in TGCT progression is also sustained by the observation from other authors, who reported in TGCT patients, an inverse correlation between progression-free survival and some circulating cytokines, including HGF [22]. To this concern, notably, c-MET availability has been related with resistance to radio- and chemo-therapy in different cancer types [23,24,25], therefore it is conceivable to hypothesize that also in TGCTs, c-MET receptor activation could lead to a refractory disease. All together these observations lead us to deeper investigate the role of c-MET-triggered signal transduction in non-seminoma cell malignant behaviour. The HGF-activated c-MET signalling pathway has been extensively studied, especially for its relationship with cancer progression [26,27,28,29]. One of the adaptors recruited by the multi-functional docking site of phosphorylated c-MET intra-cytoplasmatic tail is c-Src [30]. This protein belongs to a family of non-receptor membrane-associated tyrosine kinases (together with Fyn, Yes, Blk, Yrk, Fgr, Hck, Lck, and Lyn) [31]. It is noteworthy that c-Src is over-expressed and/or hyper-activated in a wide variety of human cancers [32,33]. Moreover, in carcinogenesis the enhanced expression or deregulation of growth factor receptors, such as c-MET, leads to c-Src over-activation that, in turn, triggers the different events that lead to the acquisition of cell malignant behaviour [34]. Based on these observations, the aim of the present study has been to shed new light on the role of c-Src in HGF-dependent and c-MET-activated aggressive behaviour of NT2D1 non-seminoma cells, studying the effects of c-Src inhibition on the already described biological responses to HGF, such as proliferation, migration, and invasion.

## 2. Results

### 2.1. c-Src is Involved in HGF-Dependent NT2D1 Cell Proliferation

**Figure 1:**
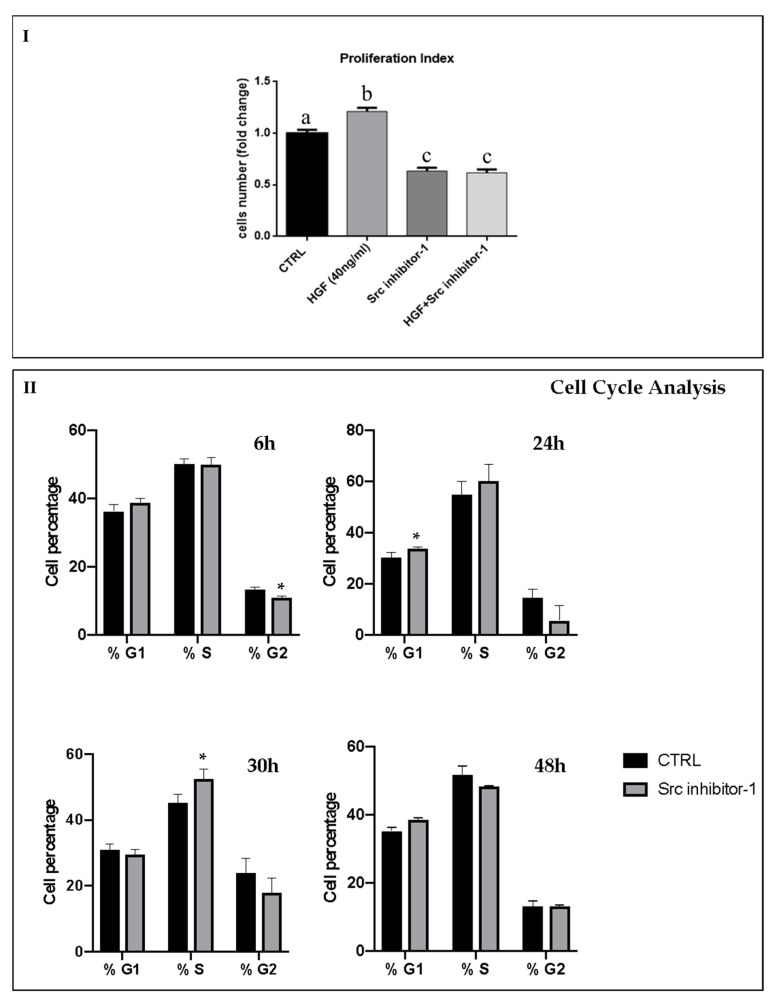
Effect of Src Inbhibitor-1 on NT2D1 cell proliferation induced by HGF. (**I**) Graphical representation of the number of NT2D1 cells cultured for 48 h in Dulbecco’s Modified Medium (DMEM) + 2% FBS alone (CTRL), or added with HGF, Src inhibitor-1, or their combination. As expected, HGF treatment shows a significant increase of cell number (b vs. a *p* < 0.001). Using the inhibitor, with or without HGF, we demonstrated a significant reduction of cell proliferation both with respect to HGF treatment (c vs. b *p* < 0.001), and to control conditions as well (c vs. a *p* < 0.001). Four independent experiments were performed at least in triplicate. Values were expressed as fold-change being the control considered arbitrarily as 1 (± SEM). (**II**) Graphical representation of cell cycle analysis on NT2D1 cell cultured for 6, 24, 30 and 48 h with or without Src inhibitor-1. (* vs. the respective CTRL condition *p* < 0.05).

We previously demonstrated that, in NT2D1 cells HGF specifically activates its receptor c-MET, triggering a significant increase of cell proliferation after 48 h of culture [21]. To investigate whether c-Src activation has a role in this phenomenon, we performed proliferation assays using an inhibitor of c-Src kinase, called Src inhibitor-1. Dose-response experiments have been carried-out in order to highlight the better working-dilution of Src inhibitor-1, and the results have been reported in Figure S1. Based on these data, we decided to use 5 µM Src inhibitor-1: this dose inhibits c-Src, at least partially (Figure S1; panel III), without triggering cell death/apoptosis (Figure S1; panel I and II). As an additional check, “trypan blue exclusion test” was performed demonstrating that the concentration of 5 µM Src inhibitor-1 does not cause cell death in this cellular line, therefore confirming that this working-dilution can be considered not toxic for this cell line. Based on the above-mentioned previous work, NT2D1 cells were cultured for 48 h in basal condition or with the following treatments: Src inhibitor-1, HGF, or HGF + Src inhibitor-1 (Figure 1). Then, cells were detached and counted. As expected, HGF administration induced a significant increase of cell number compared with control samples (1.2 ± 0.06 vs. 1 ± 0.04 respectively; *p* < 0.001). Notably, we observed that the treatment with HGF + Src inhibitor-1 completely abrogates the HGF-induced NT2D1 cell proliferation (0.7 ± 0.04 vs. 1.2 ± 0.06 respectively; *p* < 0.001). Surprisingly, using Src inhibitor-1 alone we observed a significant inhibition of cell proliferation compared with the control samples (0.7 ± 0.04 vs. 1 ± 0.04 respectively; *p* < 0.001) (Figure 1; panel I). To better characterize this phenomenon, cell cycle analyses were performed. These experiments allowed us to observe that Src inhibitor-1 administered alone causes a significant decrease of cells in G2-phase after six hours of culture, a significant increase of cells in G1-phase after 24 h of culture and a subsequent significant increase of cells in S-phase after 30 h of culture (Figure 1; panel II). These data indicate that Src inhibitor-1 causes a slight cell cycle slowdown, when administered alone. Moreover, in the light of these results, we can speculate that c-Src regulates NT2D1 cell proliferation in both HGF-dependent and HGF-independent way.

### 2.2. c-Src is Specifically Involved in HGF-Dependent NT2D1 Cell Chemoattraction

**Figure 2:**
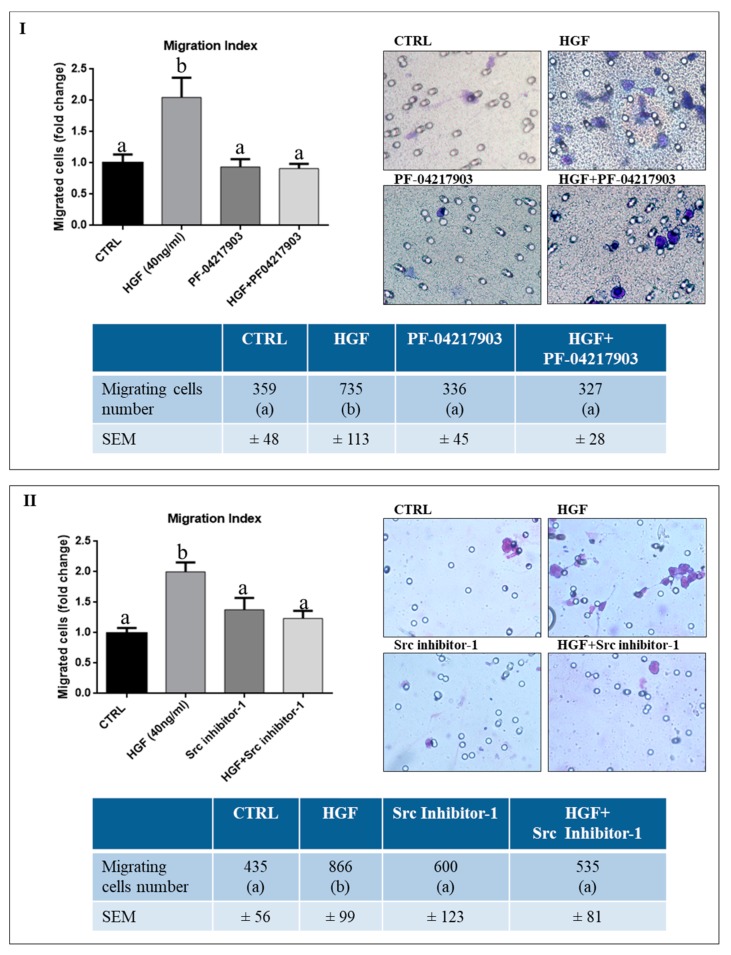
Effect of PF-04217903 and Src Inhibitor-1 on HGF-dependent NT2D1 cell chemotaxis. All experiments were performed at least in quadruplicate. (**I**) Effect of c-MET inhibitor (PF-04217903). Left panel: Graphical representation of the amount of chemo-attracted NT2D1 cells. HGF significantly increases cell migration. C-MET inhibitor (PF-04217903) administration abrogates the migratory effect induced by HGF, even if this drug is not able to affect NT2D1 cell migration when administered alone. The values of treated samples were reported as fold-change compared with control values (arbitrarily considered as 1) (b vs. a *p* < 0.001). Right panel: Representative bright-field microscopy images (40 × magnification) of the filters with the NT2D1 migrated cells. Lower panel: Table illustrating the real number of migrating cells/filter in all the experimental conditions reported in the respective “fold-change” graph (b vs. a *p* < 0.001). (**II**) Effect of Src inhibitor-1. Left panel: Graphical representation of the amount of chemo-attracted NT2D1 cells. Src inhibitor-1 abrogates the chemotactic effect induced by HGF, even if this drug is not able to affect NT2D1 cell migration when administered alone. The values of treated samples were reported as fold-change compared with control values (arbitrarily considered as 1) (b vs. a *p* < 0.001). Right panel: Representative bright-field microscopy images (40 × magnification) of the filters with the NT2D1 migrated cells. Lower panel: Table illustrating the real number of migrating cells/filter in all the experimental conditions reported in the respective “fold change” graph (b vs. a *p* < 0.001).

We previously demonstrated that HGF is a chemoattractant for NT2D1 cells [21]. To deeply investigate the specificity of this cellular response, we performed HGF-activated chemotaxis assays using the c-MET inhibitor PF-04217903 (Figure 2, panel I), as described in the “Materials and Methods” section. As expected, a significant increase of NT2D1 cell migration was observed using HGF (40 ng/mL) with respect to control condition (2 ± 0.3 vs. 1 ± 0.13 respectively, *p* < 0.001). Notably, PF-04217903 alone does not modify the migratory capability of NT2D1 cells compared with control samples (0.94 ± 0.12 vs. 1 ± 0.13 respectively, *p* = n.s.), whereas the co-administration of HGF+PF-04217903 abrogates the HGF-induced chemotactic effect (0.91 ± 0.08 vs. 2 ± 0.31 respectively, *p* < 0.001) (Figure 2, panel I). To deeper investigate the molecular effectors involved in this biological process we decided to test if c-Src is required for the HGF-mediated chemo-attraction of NT2D1 cells. We performed the above-mentioned chemotaxis assay, using Src inhibitor-1 (Figure 2, panel II). We observed that this inhibitor does not affect NT2D1 cell migration, when administered alone, compared with control samples (1.4 ± 0.2 vs. 1 ± 0.09 respectively *p* = n.s.). However, notably, the treatment with Src inhibitor-1 in the upper chamber of the trans-well apparatus specifically abrogates the chemotactic effect exerted by HGF (1.2 ± 0.13 vs. 2 ± 0.16 respectively, *p* < 0.01). Taken together these results indicate that the HGF-induced NT2D1 cell chemotaxis is specifically triggered by c-MET activation, and that c-Src is one of the adaptors directly involved in this phenomenon.

### 2.3. c-MET Modulates the Collective Migration of NT2D1 Cells Induced by HGF

**Figure 3:**
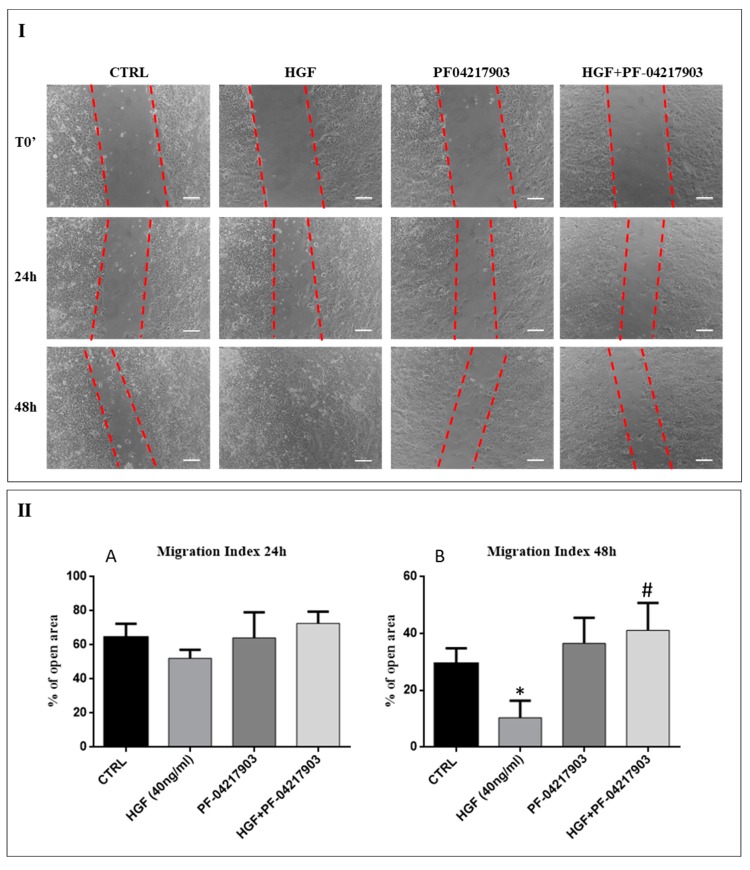
Role of HGF/c-MET system on NT2D1 cells collective motility: Wound-healing assay on NT2D1 cells cultured in DMEM + 2% FBS alone (CTRL), or added with HGF, PF-04217903, or HGF+PF-04217903. Four inserts were used for each experimental condition in each experiment. This experiment was performed in triplicate. (**I**) Representative phase-contrast images of wound-healing assay taken immediately after insert removal (T0) for wound gap (dotted red lines) measurement, or taken 24 h and 48 h after wounding. Images were photographed at 10 × magnification (scale bar: 100 µm). (**II**) Quantitative analysis of wound closure after 24 h (**A**) and 48 h (**B**) from insert removal. Data are expressed as the mean percentage of open residual area compared with the respective cell-free gap at T0. After 24 h the difference of open residual area was not statistically significant irrespectively to the experimental conditions. However, after 48 h of culture the decrease of open area in HGF treated wells was statistically significant with respect to the control wells (* *p* < 0.05). The combination of PF-04217903 + HGF abrogates the HGF-induced wound closure (# *p* < 0.01).

We analyzed in detail the effects of HGF on the collective motility of NT2D1 using the wound-healing assay performed as described in the “Materials and Methods” section. We did not observe any reduction of open residual area in the wells treated with HGF with respect to control wells after 24 h from insert removal. However, after 48 h of culture, the open residual area of HGF-treated cells resulted significantly reduced compared to the open residual area of the control wells (Figure 3; *p* < 0.05). These data demonstrate that HGF induces a significant increase of NT2D1 cell collective migration.

To evaluate the specificity of HGF/c-MET response, we performed the same experiments in presence of the specific c-MET inhibitor PF-04217903. After 24 h of culture, we did not observe any significant differences in the open residual area irrespectively from the culture condition taken into consideration (Figure 3). However, after 48 h of culture the open residual area of PF-04217903 + HGF treated wells was similar to the control wells (*p* = n.s.), but significantly higher than the open residual area of HGF treated wells (41.1% ± 9 vs. 10.4% ± 6 respectively; *p* < 0.01) (Figure 3). These data indicate that c-MET inhibition significantly reduces (about 30%) the migratory effect induced by HGF. Moreover, these results revealed that the enhancement of the HGF-triggered NT2D1 cell collective migration is specifically dependent from c-MET activation, confirming the role of c-MET in the modulation of NT2D1 cell aggressive behaviour.

**Figure 4:**
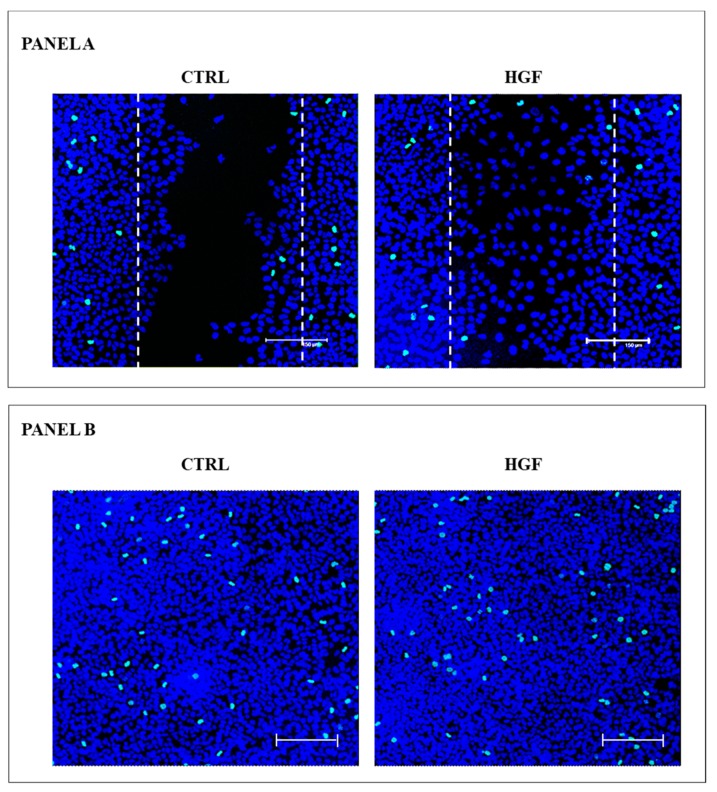
pHH3+ immunofluorescence in wound-healing assay. **Panel A**: Maximum projection of representative optical spatial series with step size of 1 µm recovered in the area of the wound. The images show NT2D1 cells, cultured for 48 h with or without HGF. Phospho-Histone H3 positive cells were immuno-labelled in green (FITC signal), whereas cell nuclei were stained with TOPRO-3 and appear blue (scale bar: 150 µm). The dotted lines indicate the presumptive area of the wound (that is 263,486 µm^2^), calculated at the beginning of the wound-healing assay. Each whole photographic-field measures 562,500 µm^2^. All experiments were performed in triplicate. **Panel B**. Maximum projection of representative optical spatial series of the same samples of Panel A recovered in areas far from the wound in which cells appear over-confluent. Each photographic-field measures 562,500 µm^2^. Phospho-Histone H3 positive cells were immuno-labelled in green (FITC signal), whereas cell nuclei were stained with TOPRO-3 and appear blue (scale bar: 150 µm).

Since HGF is able to promote NT2D1 cell proliferation, as above reported, we wanted to check whether the observed HGF-mediated increase in the rate of wound closure were due to a trigger to cell proliferation rather than to an actual migratory effect. To this end we performed immunofluorescence experiments, using the phosphohistone H3 (pHH3) as mitotic marker, on wound-healing samples at the end of their culture time. Images of pHH3 immunofluorescence were recovered both at the site of the wound, as well as in areas far from the wound (Figure 4). 

To quantify the immunofluorescence results, samples were analyzed using the Leica Confocal Software. The Sum of Fluorescence Intensity (SUM (I)) of pHH3 signal (FITC/green signal) and nuclei staining (TO-PRO3/blue signal) was calculated in three different areas of each sample. The analyzed areas were: (1) the whole photographic-field of each wound area (562,500 µm^2^ for each image); (2) the presumptive wound area (263,486 µm^2^); (3) the confluent areas recovered far from the wound (562,500 µm^2^) (Figure S2). We also counted the pHH3 positive cells in each area, and even these results are reported in Figure S2. The SUM(I) analysis as well as the count of PHH3 positive cells did not reveal any increase of pHH3 positive cell in the area in which the wound has been done, in all the samples considered. Therefore, proliferation appears to be not responsible for NT2D1 cell collective migration. It is fair to consider that even in the areas far from the wound the number of pHH3 positive cells as well as the SUM (I) of blue nuclear was not statistically significant (Figure S2). These data clearly indicate that the wound closure depends on the migratory effect of HGF on NT2D1, and that the cultural condition in which this experiment has been carried-out did not allow highlighting the HGF-mediated NT2D1 cell proliferation, probably because of the over-confluence of the cells at the plating time.

### 2.4. c-Src is Involved in HGF-Induced NT2D1 Cell Collective Migration

**Figure 5:**
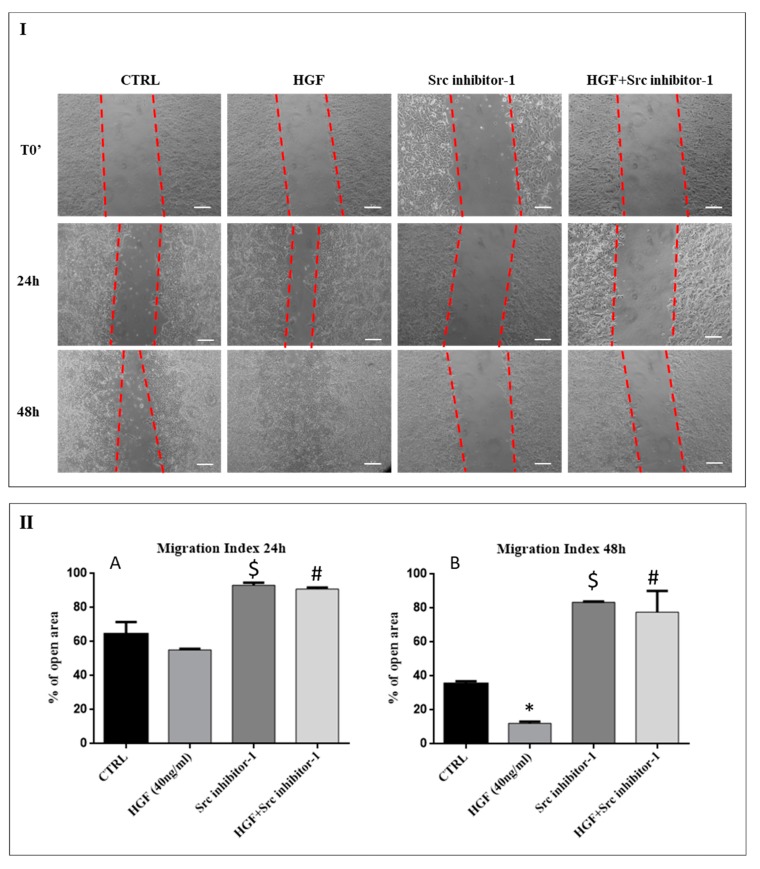
Effect of Src Inhibitor-1 on NT2D1 cell collective migration: Wound-healing assay on NT2D1 cells cultured in DMEM + 2% FBS alone (CTRL), or added with HGF, Src inhibitor-1 or HGF+Src inhibitor-1. Four inserts were used for each experimental condition in each experiment. The experiment was performed in triplicate. (**I**) Representative phase-contrast images of wound-healing assay taken immediately after insert removal (T0) for wound gap (dotted red lines) measurement, or taken 24 h and 48 h after wounding. Images were photographed at 10 × magnification (scale bar: 100 µm). (**II**) Quantitative analysis of wound closure after 24 h (**A**) and 48 h (**B**) from insert removal. Data are expressed as the mean percentage of open residual area compared with the respective cell-free gap at T0. After 24 h the difference of open residual area between CTRL and HGF treated wells was not statistically significant. However, after 48 h of culture the decrease of open area in HGF treated wells was statistically significant with respect to the control wells (* *p* < 0.001). Src inhibitor-1 in combination with HGF both at 24 h (# *p* < 0.01) and 48 h (# *p* < 0.001) abrogated the migratory effect induced by HGF. Src inhibitor-1 alone was also able to inhibit the basal collective migration of the NT2D1 cells, compared with control wells, both at 24 h ($ *p* < 0.05), and 48 h ($ *p* < 0.001) after insert removal.

It is well known that HGF binding activates c-MET, resulting into tyrosine residues phosphorylation. Activated c-MET recruits signaling effectors that activate protein kinase, such as c-Src, ultimately leading to cell migration [20]. To investigate whether collective migration induced by HGF treatment would depend on c-Src recruitment of c-MET signaling cascade, wound-healing assays were performed treating NT2D1 cells with HGF with or without the Src inhibitor-1 (Figure 5). As already mentioned, the dose of Src inhibitor1 used in our experiments does not alter NT2D1 cell viability (Figure S1).

The results shown in Figure 5, confirm that HGF is able to enhance NT2D1 cell collective migration in 48 h of culture. This part of the results obtained, substantially overlap with those reported in Figure 3. The treatment with Src inhibitor-1, alone or in combination with HGF, gave very interesting results, strongly indicating a role of this adaptor in NT2D1 cell collective migration.

More in detail, after 24 h of culture the open residual area of samples treated with Src inhibitor-1 alone showed values similar to its own T0 (93.1% ± 1.5) and, therefore, significantly higher compared with control condition (64.4% ± 7.1 *p* < 0.05) and HGF treated wells (55.2% ± 0.6 *p* < 0.05). At 48 h these areas remained almost unchanged (83.5% ± 0.3) with respect to 24 h, whereas the open residual area of control samples and HGF treated wells decreased at level of 35.4% ± 1.5 and 12.2% ± 1 respectively (Figure 5). The inhibition of collective cell migration in the presence of Src inhibitor-1 alone clearly indicates the involvement of this adaptor-protein in constitutive collective migration of NT2D1 cells, even if further investigations are needed to clarify this point.

Src inhibitor-1 administration reduces also the enhanced collective migration induced by HGF. The values of open residual area of HGF+Src inhibitor-1 treated wells were significantly higher, compared with HGF treated wells, already after 24 h of culture (90.9% ± 0.9 vs. 55.2% ± 0.6 respectively; *p* < 0.05). This difference was even more significant after 48 h of culture (77.6% ± 12 vs. 12.2% ± 1 respectively; *p* < 0.001) (Figure 5).

### 2.5. c-Src is Involved in HGF-Dependent NT2D1 Cell Invasion

**Figure 6:**
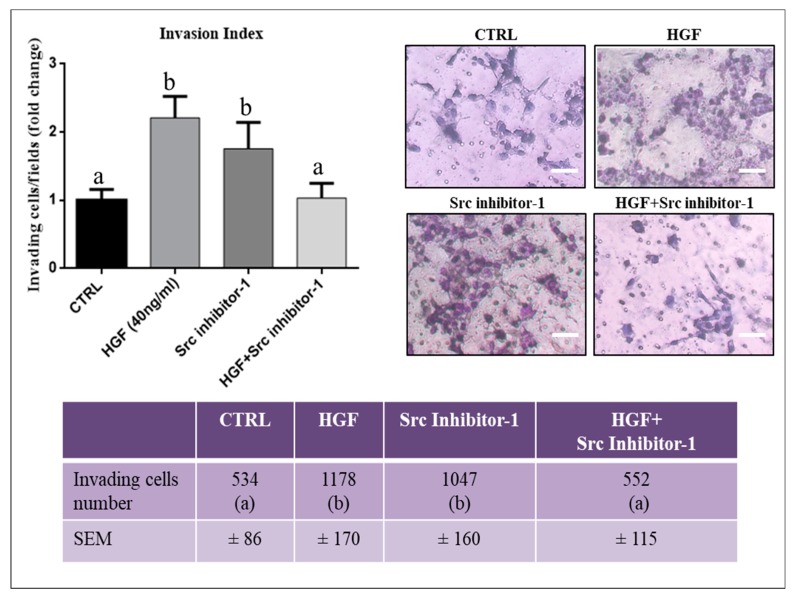
Effect of Src Inhibitor-1 on NT2D1 cell invasiveness: Invasion assay was performed using filters coated with GFR-Matrigel. The filters were analyzed by optical microscope. Three independent experiments were performed at least in triplicate. Left panel: Quantitative analysis of invading cells. The results are expressed as fold-change (± SEM) and the control condition has been arbitrarily considered as 1. Both HGF and Src inhibitor-1, administered alone, induce a statistically significant increase of NT2D1 cell invasion with respect to control samples (b vs. a, *p* < 0.05). The co-administration of HGF+ Src inhibitor-1, abrogates the HGF-dependent NT2D1 cell invasion. Right panel: Representative bright-field microscopy images of the different culture conditions (scale bar: 70 µm). Images demonstrate a higher invasive behaviour of cells treated with HGF or Src inhibitor-1 alone compared with HGF + Src inhibitor-1 or control conditions. The table in the lower panel illustrates the real number of invading cells/filter in all the experimental conditions reported in the respective “fold change” graph (b vs. a, *p* < 0.05).

To better understand the mechanism underlying the HGF-dependent NT2D1 cell invasion, already reported by our group [21], we performed invasion assays on Matrigel coated filters, using the Src inhibitor-1. As shown in Figure 6, we confirm that HGF treatment significantly enhances the invading capability of NT2D1 cells (2.2 ± 0.3 vs. 1 ± 0.16 respectively; *p* < 0.05). When Src inhibitor-1 was used in combination with HGF the number of invading cells reverts to control condition (1 ± 0.21 vs. 1 ± 0.16 vs. respectively *p* = n.s.). Interestingly, we also found that Src inhibitor-1 administered alone, significantly increased NT2D1 cell invasiveness compared with control condition (1.75 ± 0.4 vs. 1 ± 0.16 vs. respectively *p* < 0.05) at similar level to that obtained after HGF administration (2.2 ± 0.3 vs. 1.75 ± 0.4; *p* = n.s.) (Figure 6). This result is relevant and deserves further investigations to characterize in detail the biological mechanism underlying it. Taken together, these data demonstrate that c-Src is involved in the regulation of NT2D1 cell invasiveness using both HGF-dependent and HGF-independent pathways.

### 2.6. Phospho-c-Src Detection after HGF Administration to NT2D1 Cells

**Figure 7:**
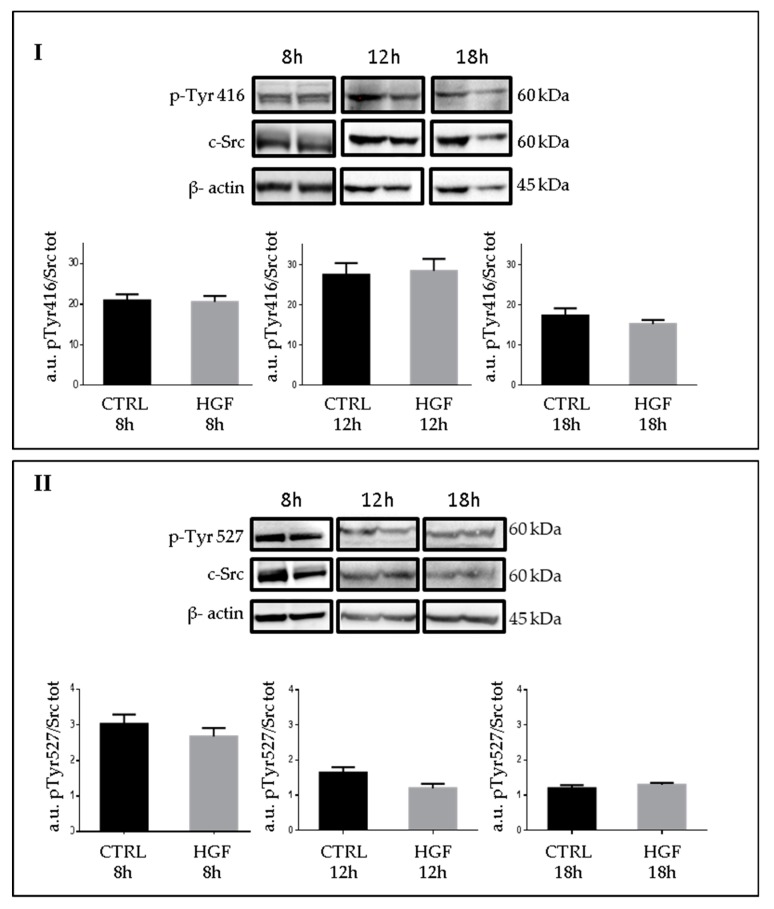
c-Src expression and phosphorylation of NT2D1 cells exposed to HGF: (**I**) Western blot analyses of c-Src Phospho Tyr 416, total c-Src protein, and β- actin in NT2D1 cell lines cultured for 8 h, 12 h and 18 h with or without HGF. In the lower part of the panel the densitometric analyses of c-Src phospho Tyr 416 bands normalized versus total c-Src/β-actin are shown. (**II**) Western blot analysis of c-Src phospho Tyr 527, total c-Src protein, and β- actin in NT2D1 cell lines cultured for 8 h, 12 h and 18 h with or without HGF. In the lower part of the panel the densitometric analyses of c-Src phospho Tyr 527 bands normalized versus total c-Src/β-actin are shown.

It is known that c-MET phosphorylation determines several biological responses, and in a previous work, we demonstrated that HGF triggers c-MET phosphorylation, in NT2D1 cells, 6 h after treatment [21]. To analyze whether c-Src phosphorylation changes after HGF administration we performed western blot analyses at different culture times (8, 12 and 18 h) (Figure 7). C-Src activation is peculiar since it is regulated by the phosphorylation of two key tyrosines: Tyr527 that needs to be dephosphoryled to allow c-Src activation that is in turn due to the autophosphorylation of Tyr416. Therefore, we performed western blot analyses using anti-c-Src Phospho Tyr 416 (active c-Src), and anti-c-Src Phospho Tyr 527 (inactive c-Src), both normalized versus total c-Src/β actin. Surprisingly, we did not observe significant modulation of c-Src active or inactive isoform due to HGF administration. Notably, fluctuation of both isoforms is reportable during culture time, indicating that a constitutive turnover of activation/inactivation occurs (Figure 7).

### 2.7. Immunofluorescence Analysis of the Active Form of c-Src (Phospho Tyr 416) in NT2D1 Cells

**Figure 8:**
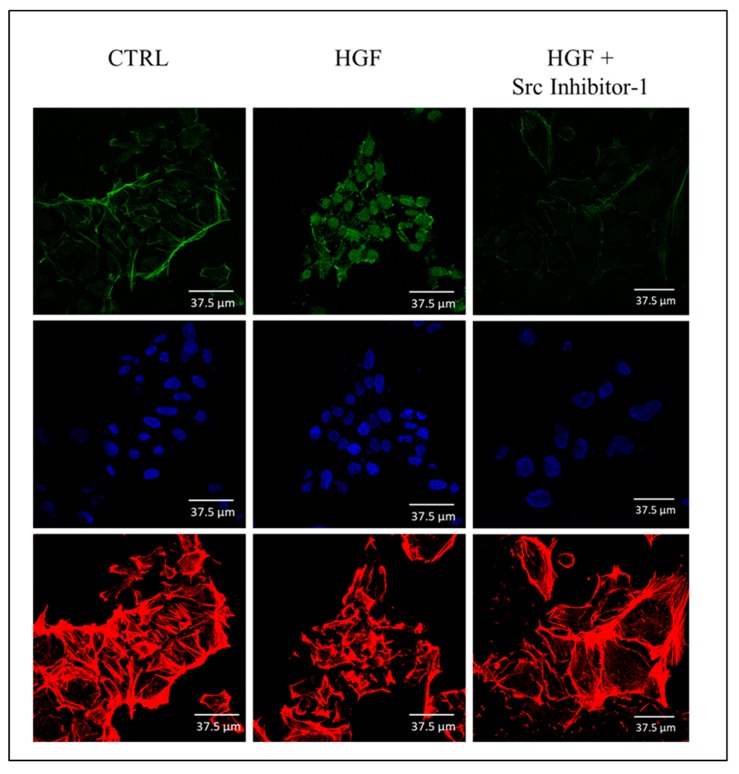
Confocal microscopy analysis of c-Src phospho Tyr416 distribution-pattern: Immunofluorescence analyses of c-Src phospho Tyr416 (FITC, green signal) in NT2D1 cells cultured with or without HGF and Src Inhibitor-1. Actin cytoskeleton (Rhodamin phalloidin, red signal) and nuclei staining (TOPRO-3, blue signal) are also reported in the figure. The nuclear translocation of c-Src phospho Tyr416 after HGF administration is clearly evident. The treatment with Src Inhibitor-1 prevents this phenomenon.

To describe the distribution-pattern of the active form of c-Src in our experimental conditions, we performed immunofluorescence analyses using anti-c-Src (phospho Tyr 416). Interestingly, we found that, after HGF administration the active form of c-Src appears mainly localized in the nucleus, even if the cytoplasmic/membrane localization does not disappear in the HGF treated samples (Figure 8). The presence of c-Src in the nuclear compartment of normal and cancer cells has been already reported by some groups [35,36,37] even if the relevance of this phenomenon for cancer cell aggressive behaviour gave, so far, discordant results. In our experimental model, this observation triggers the intriguing hypothesis that HGF-mediated c-Src activation caused rapidly the modification of gene expression via the translocation of the active form of c-Src in the nucleus of NT2D1 cells. Noteworthy, when Src inhibitor-1 is co-administered with HGF, nuclear translocation of c-Src is prevented (Figure 8).

## 3. Discussion

As mentioned in the introduction section, all type II TGCTs arise from a common precursor lesion, called GCNIS, though their neoplastic evolution can be very variable. Seminoma and non-seminomas are characterized by a very different histology in fact: seminomas appear as homogenous tumors that resemble undifferentiated gonocytes, whereas non-seminomatous lesions are more heterogeneous. Actually, a genetic reprogramming of GCNIS cells occurs at the onset of non-seminoma lesion, giving rise to Embryonal Carcinoma, a malignant caricature of embryonic stem cells. Non-seminomas are generally more aggressive than seminomas and have a younger age of diagnosis [38]. The main clinical concerns of these cancers (that are mostly chemo- and radio-sensitive), is represented by: (1) the resistance to chemotherapy of a fraction of them [39], (2) the long-term morbidity associated with the use of the chemotherapeutics [38]. Even if, several Genome-Wide Association Studies (GWAS) revealed important loci variants in TGCTs [26,27,28,40,41,42,43,44,45,46], very few targetable mutated genes have been discovered so far, in spite of the high aneuploidy that features these cancer types [47]. Therefore, the possibility of target-drug based therapies on TGCTs is still questionable and under investigation [39,48,49,50]. It is worth mentioning, as reported in the introduction section, that it is commonly accepted that the onset and progression of TGCTs depends on the testicular niche, and therefore even on the Growth Factors and Growth Factor-activated pathways present in the neoplastic testicular microenvironment. As emerged from literature data, an even more detailed study of this aspect could represent a good starting-point for the establishment of more personalized therapies even in order to address the problem of refractory diseases [22,47,50]. In a recent paper we found that, HGF is able to stimulate the malignant and aggressive behaviour of NT2D1 non-seminoma cells, and that this phenomenon depends on c-MET activation [21]. c-MET signaling pathway is rather complex, since phosphorylated c-MET tail contains a multifunctional docking site for several SH2-containing signal transducers, whose coordinated actions drive different cell activities such as, proliferation, polarized and collective migration and invasion [51]. c-Src is one of these transducers, whose action has been extensively studied in tumorigenesis and metastatic progression [52]. c-Src, appears over-expressed or over-activated in a wide variety of cancers, in fact [32]. Moreover, it is recruited by several tyrosine kinase receptors of the Growth Factors considered as neoplastic micro-environmental cues [31,53,54,55]. Notably, c-Src is recruited also by c-KIT receptor that is the prominent factor considered involved in the onset of testicular germ cell tumorigenesis [47]. Moreover, c-Src activation, in several signaling pathways, triggers in turn the activation of RAS transducer, and this is one of the few proteins, together with c-KIT receptor, whose gene appears mutated in a significant number of patients affected by TGCTs [47]. In the light of these considerations, we decided to study the role of c-Src in the HGF-dependent and c-MET-activated NT2D1 cell activities that emerged in our previous work [21]. In our experimental model, c-Src inhibition abrogates the HGF-dependent increase of cell proliferation, polarized and collective migration as well as cell invasion. Notably, the administration of c-Src inhibitor alone does not affect NT2D1 cell basal migration (HGF-independent) in chemotaxis assays, indicating that this adaptor protein, in our experimental conditions, is specifically recruited by c-MET signaling pathway to trigger this cellular response. Surprisingly, c-Src inhibition in basal culture conditions increases NT2D1 invasiveness and, at the same time, decreases the cell proliferation rate and collective migration capability of NT2D1 cells independently from c-MET pathway activation. Therefore, from the results presented here it is possible to hypothesize that c-Src is recruited by c-MET signaling pathway when this receptor is activated by HGF, but that c-Src is also recruited by housekeeping homeostatic pathways that balance the aggressive behaviour of NT2D1 cells. To this regard, it is fair to highlight that Selfe and co-workers [49] recently revealed a panel of tyrosine kinase receptors constitutively phosphorylated in NT2D1 cells. Therefore, we can speculate that, some of the constitutively activated signaling pathways could recruit c-Src. In line with this hypothesis, the data presented herein reveal that c-Src phosphorylation appears constitutively present in NT2D1 cells: the active and inactive forms are both detectable in these cells even when cultured in basal conditions, and they are not significantly modulated by HGF administration. However, immunofluorescence analyses revealed that HGF treatment triggers the translocation of c-Src active form into the nucleus, indicating a recruitment of this adaptor-protein in the direct modulation of cellular transcription downstream c-MET signaling pathway activation. Intriguingly, the nuclear c-Src (phospho Tyr 416) has been demonstrated to interact with the promoter of specific genes that modulate pancreatic cancer cell migration [35,36,37]. Taken together these observations are biologically relevant and lead us to consider the importance of micro-environmental cues present in the tumoral niche for the anti-tumoral response to target-therapies. For this reason, in our opinion, a detailed description of the TGCTs micro-environmental cues can represent a fundamental prerequisite to address a more personalized treatment of TGCTs neoplasms. Moreover, the results herein reported strongly stimulates to investigate deeply, at proteomic level, the overexpression of HGF as well the over-activation of c-MET and c-Src in TGTCs biopsies with special focus on non-seminoma lesions.

## 4. Materials and Methods

### 4.1. Cell Culture

NT2D1 embryonal carcinoma cell were purchased from the ATCC. This cell line was cultured in DMEM (Sigma Aldrich, cat. D6546, St Louis, MO, USA) supplemented with 10% Foetal Bovine Serum (FBS Gibco, cat. 10270, Gland Island, NY, USA), L-Glutamine (Sigma Aldrich, cat. G7513) and penicillin/streptomycin (Sigma-Aldrich, cat. P0781). The cells were used from passage 15 to 35. Mycoplasma testing was routinely done with the N-GARDE Mycoplasma PCR Reagent set (Euro-Clone, cat. EMK090020, Milano, Italy).

The cells were treated with 40 ng/mL of HGF (Human Recombinant HGF, R&D Systems, cat. 294-HG, Minneapolis, MN, USA), 50 µM of a c-MET specific inhibitor (PF04217903; Sigma-Aldrich, cat. SML0263), or 5 µM of a Src inhibitor (Src Inhibitor-1; Sigma-Aldrich, cat. S2075). 

We tested different concentrations (1 µM to 10 µM) of Src inhibitor-1 on cell viability. 5 µM, was the highest concentration without toxic effect evaluated by trypan blue exclusion test, while, PF04217903 was used as already described in a recent work [21].

### 4.2. Cell Proliferation Assay

To test the role of c-Src on HGF-induced NT2D1 cell proliferation we performed proliferation assays using the c-Src inhibitor-1. To this end, we cultured 9 × 10^4^ NT2D1 cells cultured in 12-well plates in DMEM 10% FBS. After 24 h, the cells were starved for 16 h under serum-free conditions and then were treated with DMEM 2% FBS (control condition), adding, when indicated, Src inhibitor-1, HGF, or Src inhibitor-1+HGF. After 48 h, cells were trypsinized, harvested, and counted. Each experiment was performed at least in triplicate. Four independent experiments were performed. The results (Mean ± SEM) are expressed as fold-change considering control condition as 1.

### 4.3. Cell Cycle FACS Analysis

Cells were seeded at 5 × 10^5^ cells in 60 mm petri dishes. After 24 h cells were maintained under serum-free conditions for 16 h (starvation). Then, cells were cultured for different times (6, 24, 30 and 48 h) in the absence or in the presence of Src inhibitor-1 (5 µM) in 2% FBS. The cells were harvested, fixed in 70% EtOH at 4 °C, and stained with propidium iodide (50 μg/mL)/RNase (100 U/mL) solution (Sigma- Aldrich, cat. P4864 and R6513 respectively) for at least 3 h. The cell suspensions were analyzed with CyAn ADP (Beckman Coulter, Fullerton, CA, USA) and data were analyzed with the FCS Express 5.1 software (De Novo, Los Angeles, CA, USA).

### 4.4. Cell Death Assay

Cells, maintained under serum-free conditions for 16 h, were treated with Src inhibitor-1 at different concentrations (1, 2.5, 5 and 10 µM) for 48 h or only at 5 µM for 72 h. Since Src inhibitor- is soluble in Dimethyl Sulfoxide (DMSO), the respective dose of DMSO alone was added to control samples. Cell death was evaluated with propidium iodide exclusion assay, using 2 µg/mL of propidium iodide (Sigma- Aldrich, cat. P4864). Cell death was evaluated by flow cytometry using CyAn ADP (Beckman Coulter), and data were analyzed with the FCS Express 5.1 software (De Novo).

### 4.5. Chemotaxis Assay

Chemotaxis assay were performed using the Cell Culture Inserts (12 well 8.0 µm pore size; Falcon, cat. 353182, Lincon Park, NJ, USA) placed in 12-well culture plates (Falcon, cat. 351143). Cells were starved for 16 h under serum free conditions. Part of the cells were pre-treated with DMEM 10% FBS+PF-04217903 for 30 min, to inhibit c-MET phosphorylation, or with DMEM 10% FBS + Src inhibitor-1 for 48 h, to inhibit c-Src phosphorylation. Then, cells were trypsinized, counted, and resuspended in DMEM without serum; 2 × 10^5^ cells/well in 1.4 mL DMEM were added in the upper chamber of the trans-well, in absence (DMEM alone), or in presence of PF-04217903, or Src inhibitor-1. The lower chambers were filled with 800 µL DMEM alone (negative control) or DMEM+HGF. Cells were incubated at 37 °C with 5% CO_2_. After 5 h, the medium and un-migrated cells, in the upper surface of the insert, were mechanically removed, and the lower surface of the insert, containing migrated cells, was fixed with paraformaldehyde 4% in PBS (pH 7.4) at 4 °C, and stained with Diff Quick solution (DADE, cat. 130832, Network, NJ, USA). Migrated cells were visualized under 40 × objective using a bright-field Optical Microscopy (Axioplan, Zeiss, Oberköchen, Germany). For cell counting, each filter has been divided in sectors and all sectors of each filter have been photographed under the 40 × magnification objective and counted. In this way, we considered to have approximately counted the whole area of each filter. The migrated cells were counted manually, with the help of ImageJ software. Three independent experiments were performed. Each experiment was performed at least in quadruplicate. The results (Mean ± SEM) are expressed as fold-change being control condition arbitrarily considered as 1.

### 4.6. Matrigel Invasion Assay

For assessment of invasion, in vitro invasion assay was performed using chambers coated with GFR-Matrigel (Basement Membrane Matrix Growth Factor Reduced, BD Biosciences, cat. 354483, San Jose, CA, USA). Cells were starved for 16 h in DMEM without FBS and then were pre-treated with Src inhibitor-1 for 48 h. Cells were then trypsinized, counted and resuspended; 2.5 × 10^4^ cells/well in 500 µL DMEM 2% FBS were seeded on the top of the GFR-Matrigel (control condition). When indicated Src inhibitor-1, HGF, or Src inhibitor-1+HGF were added to cell suspension. The lower chambers were filled with 750 µL DMEM supplemented with 2% FBS. Cells were incubated for 24 h at 37 °C with 5% CO_2_ and then GFR-Matrigel and non-invading cells were mechanically removed with cotton swabs. Filters containing invading cells were fixed with paraformaldehyde 4% in PBS (pH 7.4) at 4 °C, and stained with Diff Quick solution. The filters were analyzed by bright field Optical Microscopy and four fields per filter were photographed at 10 × magnification to obtain a global view of cell invasion. For cell counting, each matrigel-coated filter has been divided in sectors and all sectors of each filter have been photographed under the 40 × magnification objective and counted. In this way, we considered to have approximately counted the whole area of each filter. The migrated cells were counted manually, with the help of ImageJ software. Invading cells were counted and the average number ± SEM of cells are reported as fold change being control considered as 1. Three independent experiments were performed, and each experiment was performed at least in triplicate.

### 4.7. Wound-healing Assay (Collective Migration Assay)

To perform the wound-healing assay, we used special double well culture inserts (Ibidi GmbH, Martinsried, Germany). Each insert was placed in a well of a 24-well plate. Cells were starved for 16 h under serum free conditions, then they were detached, and 3.5 × 10^4^ cells were placed into both wells of each insert with 70 μL of medium containing 2% FBS. When cells are confluent, the culture inserts were gently removed, and cells were fed with 2% FBS DMEM (CTRL), or treated with HGF (40 ng/mL), PF-04217903 (50 μM), HGF+PF-04217903, Src inhibitor-1 (5 μM), HGF+ Src inhibitor-1, all diluted in CTRL medium. Each well was photographed at 10 × magnification immediately after insert removal, for the measurement of the wound (cell-free) area (T0 area considered as 100%), and after 24 h and 48 h with a Nikon DS-Fi1 camera (Nikon Corporation, Tokyo, Japan), coupled with a Zeiss Axiovert optical microscope (Zeiss, Oberkochen, Germany). The mean percentage of residual open area compared with the respective cell-free space taken at T0 was calculated using ImageJ v 1.47 h software. For each experimental condition, four independent experiments were performed in triplicate.

### 4.8. Immunofluorescence Analyses

To describe the distribution pattern of the active form of c-Src, Cleaved Caspase-3 and pHH3, immunofluorescence experiments, followed by Confocal Microscopy analysis, were performed. Briefly, cells were fixed in paraformaldehyde 4% in PBS (pH 7.4) at 4 °C for 15 min, and permeabilized in PBS supplemented with 1% BSA and 0.1% Triton for 2 h. Then samples were incubated overnight with one primary antibody of the followings: anti-c-Src (phospho Tyr 416) antibody (GeneTex n. GTX 81151; 1:25 dilution), anti-cleaved Caspase-3 antibody (Cell Signaling D175; 1:80 dilution), and anti-phospho-histone H3 antibody (pHH3 mouse monoclonal, Santa Cruz, cat. sc-374669, CA, USA, 1:50 dilution). Then, samples were washed three times in PBS/BSA/Triton for 30 min, and incubated with the appropriate secondary antibody: FITC-conjugated donkey anti-rabbit IgG (Jackson Immuno Research, cat. 711-095-152, West Grove, PA, USA, dil. 1:200) for phospho c-Src and Cleaved Caspase-3 detection, and FITC-conjugated donkey anti-mouse IgG (Jackson Immuno Research, cat. 715-095-150, dil. 1:200) for pHH3 detection. Immunofluorescence experiments were analyzed using Leica Confocal Microscope (Laser Scanning TCS SP2 equipped with Kr/Ar and He/Ne lasers, Mannheim, Germany). TO-PRO3 iodide fluorescent dye 642/661 (1:5000 in PBS, Invitrogen, cat. T3605, Carlsbad, CA, USA) for nuclei staining and rhodamine phalloidin (Invitrogen Molecular Probes Eugene 1:40 dilution) for F-actin visualization were used. Quantitative analysis, (Sum of Intensity (SUM (I)) of Phospho-Histone H3 positive cells (FITC/green signal) and nuclei (TOPRO-3/blue signal) was performed by Leica Confocal software. 

### 4.9. Western Blot Analyses

The cells were cultured for 8, 12 and 18 h in DMEM 2% FBS with or without HGF. At the end of this culture time, cells were solubilized in lysis buffer (1% SDS, 10 mM Tris, pH 7.5) containing protease and phosphatase inhibitors (Roche, cat. 04693124001 and 04906837001, Mannheim, Germany). Protein concentration was determined using the BCA protein assay (Pierce, cat. 23221), and 40 μg/lane were loaded into 7% SDS–PAGE under reduction condition. Precision Plus Protein All Blue Standards (Bio-Rad Laboratories, Hercules, CA, USA) were used as molecular weight markers. Proteins were electro-transferred to nitrocellulose membranes (Bio-Rad, cat. 1620115). Membranes were blocked through incubation with 5% BSA (Sigma-Aldrich, cat. A2153) in TBS-T buffer (20 mM Tris, pH 7.6, 150 mM NaCl, 0.1% Tween-20), to avoid non-specific binding of the antibodies. Then, the membranes were incubated with anti-c-Src (all isoforms) antibody, raised in rabbit (1:1000, Cell Signaling, cat. 2108), or anti-c-Src (phospho Tyr 416) antibody (GeneTex n. GTX 81151; San Antonio, TX, USA, 1:100 dilution), or anti-c-Src (phospho Tyr 527) antibody (GeneTex n. GTX 50210; 1:500 dilution). Primary antibodies were incubated for 16 h at 4 °C. For the detection, nitrocellulose membranes were incubated with HRP conjugated secondary antibody Anti-Rabbit IgG (1:5000, GE Healtcare UK Limited, Buckinghamshire, England, cat. NA9340V). To normalize total protein extracts, monoclonal anti-β-actin directly conjugated with HRP (1:10,000, Sigma-Aldrich, cat. A3854) was used. For the band visualization, the ECL western blotting detection reagent, (Euroclone, cat. EMP011005) was used. Membranes were analysed by ChemiDoc XRS and the image acquired were processed by Image Lab software (Bio-Rad Laboratories). Phospho c-Src densitometric profiles have been normalized versus total c-Src/β actin. Three independent experiments were performed, and each experiment was performed at least in duplicate.

### 4.10. Statistical Analyses

All quantitative data are presented as the mean ± standard error (SEM). The statistical analyses have been carried-out using Sigma Plot 11 Data Analyzer Software. Student’s t-test and ANOVA test (for multi-group comparison) were carried-out. Data-Analyzer Software chose the ANOVA’s post-hoc tests, depending on data distribution. In detail, Fisher’s Least Significant Difference (LSD) has been applied to all the ANOVA tested experimental data, except for wound-healing data that were analyzed by Dunn’s test. All experiments were performed at least in triplicate, and the significance level was fixed at a *p* value < 0.05.

## Supplementary Materials

Supplementary materials can be found at http://www.mdpi.com/1422-0067/20/2/320/s1.

## Abbreviations

BCA	Bicinchoninic Acid assay
BSA	Bovine Serum Albumine
c-MET	Mesenchymal Epithelial Transition factor (HGF receptor)
FBS	Foetal Bovine Serum
GCNIS	Germ Cell Neoplasia In Situ
GFR	Growth Factor Reduced
HGF	Hepatocyte Growth Factor
SEM	Standard Error Measure
SUM (I)	Sum of Intensity
TGCTs	Testicular Germ Cell Tumours
