# Supplementary material for: c-Src Recruitment is Involved in c-MET-Mediated Malignant Behaviour of NT2D1 Non-Seminoma Cells
Source: Int J Mol Sci. 2019 Jan 14;20(2):320. doi: 10.3390/ijms20020320 (PMC6358843; doi:10.3390/ijms20020320)
Supplement: Supplementary file 1 [file ijms-20-00320-s001.pdf]

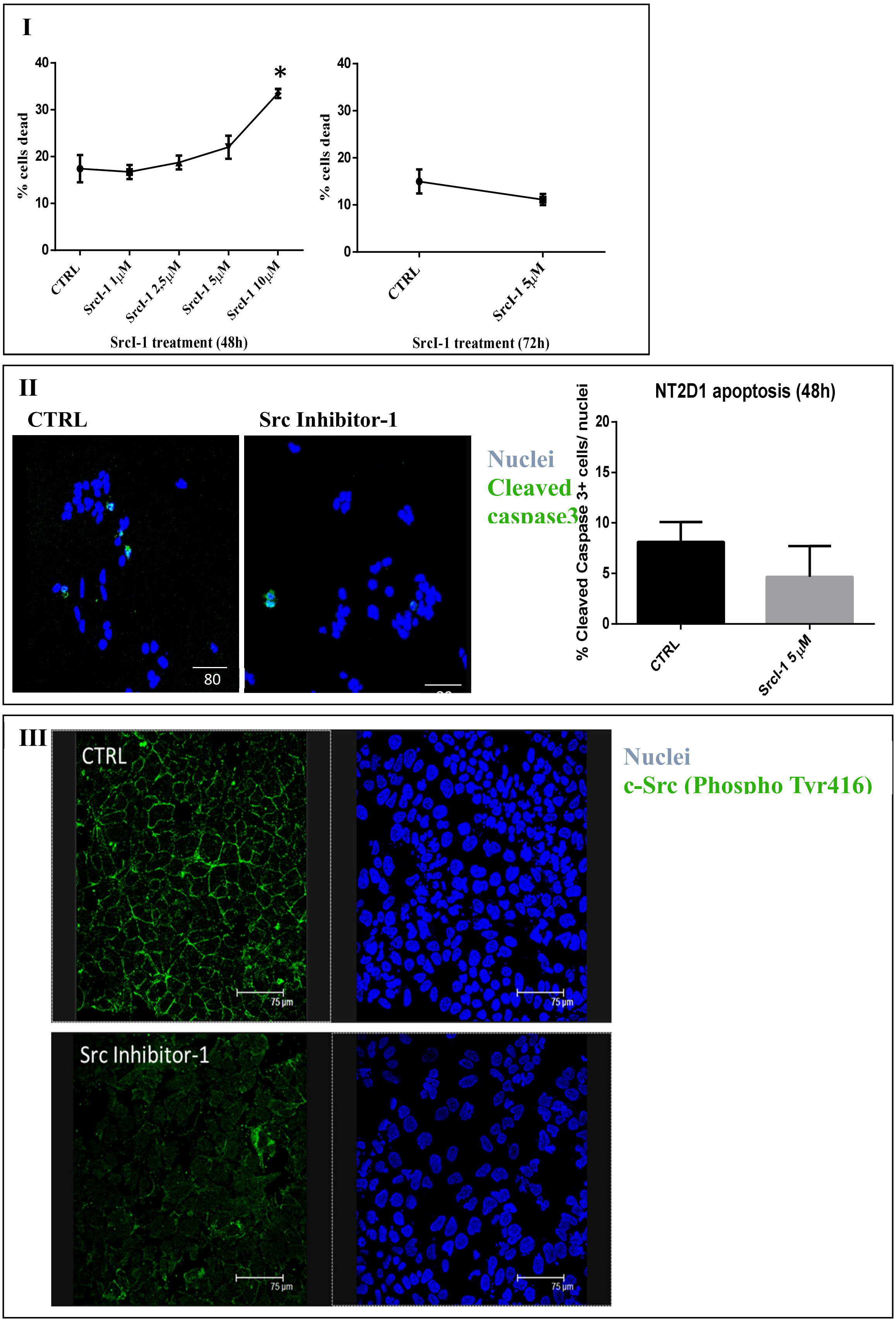

Figure S1. Panel I Cell death FACS analysis on NT2D1 cells. On the left, the graphical representation of the percentage of dead cells obtained culturing NT2D1 cells with different concentration of Src inhibitor-1 (1, 2.5, 5, 10 μM) for 48h is shown. It is evident that 10 μM Src inhibitor-1 causes a significant increase in the percentage of cell death compared with control condition ($p<0,05$). On the right the graphical representation of the

percentage of dead cells, obtained culturing for 72h NT2D1 cells with 5 μM Src inhibitor-1 is shown. **Panel II** Representative images of cleaved caspase3 immunofluorescence on cells treated with 5 μM Src inhibitor-1 for 48h (scale bar 80 μM). On the right the quantitative analysis, by Leica Confocal analysis of the number/field of cleaved caspase3 positive cells (FITC/green signal) normalized versus and the number of nuclei (TOPRO-3/blue signal) is shown. **Panel III** Representative images of immunolocalization of phospo c-Src 416 in NT2D1 cells cultured for 48h with or without 5 μM of Src inhibitor-1 (scale bar 75 μM). The images reveal that Src inhibitor-1 treatment decreases the signal for the active form of c-Src, but does not abolish it.

PANEL A

Confocal analysis of the whole field in the wound areas			
Table 1	SUM (I) green	SUM (I) blue	pHH3+ cells
CTRL	1359 ± 300	63011 ± 2584	19 ± 2,8
HGF	1800 ± 265	65253 ± 4502	22 ± 1,1
p value	n.s.	n.s.	n.s.

Confocal analysis of the wound area only (central part of the field)			
Table 2	SUM (I) green	SUM(I) blue	pHH3+ cells
CTRL	230,739 ± 25,037	20636 ± 1629	4 ± 0,4
HGF	308,278 ± 36,800	32167 ± 955	4,5 ± 0,6
p value	n.s.	P<0,001	n.s.

PANEL B

Confocal analysis of the areas far from the wound			
Table 3	SUM (I) green	SUM (I) blue	pHH3+ cells
CTRL	5733 ± 780	915604 ± 154953	53 ± 8
HGF	6559 ± 970	933214± 164504	67 ± 6
p value	n.s.	n.s.	n.s.

Figure S2. Confocal microscopy quantitative analysis of the sum of fluorescence intensity (SUM(I)) of Phospho-Histone H3 positive cells and nuclei in wound-healing assay.

Panel A. Table 1: Quantitative analysis, by Leica Confocal software, of Phospho-Histone H3 positive cells (FITC/green signal) and nuclei (TOPRO-3/blue signal) calculated in the whole photographic field of the wound areas (562500 μm^2 for each image) reported in Figure 4 of the manuscript. Phospho-Histone H3 positive cells per field have been also counted. This analysis takes into consideration both the central parts (wound area) and the lateral parts of each image. The values reported in this table clearly show that there is no significant difference in the amount of Phospho-Histone H3 positive cells between control and HGF treated cells. **Table 2:** Quantitative analysis, by Leica Confocal Software, of Phospho-Histone H3 positive cells and nuclei calculated in the central part of the images (inside the dotted lines of the representative images of panel A),

that is the presumptive wound area (263486 μm^2) reported in Figure 4 of the manuscript. Phospho-Histone H3 positive cells per field have been also counted. The values reported in this table showed that there is no significant difference in the amount of Phospho-Histone H3 positive cells between control and HGF treated cells, even in the areas of the wound. Notably, a significant increase of blue fluorescence (that represents nuclear staining) is observable between control condition and HGF treated cells. All together these observations indicate that HGF stimulates the collective migration of NT2D1 cells, and that, in this cultural conditions, HGF-triggered cell proliferation does not contribute significantly to wound closure.

Panel B. Table 3: Quantitative analysis, by Leica Confocal software, of Phospho-Histone H3 positive cells (FITC/green signal) and nuclei (TOPRO-3/blue signal) calculated in the whole photographic field (562500 μm^2 for each image) reported in Figure 4 of the manuscript. Phospho-Histone H3 positive cells per field have been also counted. The reported values showed that, that there is no significant difference in the amount of Phospho-Histone H3 positive cells between control and HGF treated cells, even in the confluent part of the culture dishes.
